# Learning About Healthy Nutrition by Doing: Experiential Approaches in School-Based Nutrition Education

**DOI:** 10.3390/nu18101610

**Published:** 2026-05-19

**Authors:** Arianna Bisogno, Ludovica Leone, Veronica D’Oria, Carlo Agostoni, Martina Abodi

**Affiliations:** 1 Pediatric Unit, Foundation IRCCS Ca’ Granda Ospedale Maggiore Policlinico, 20122 Milan, Italy; arianna.bisogno@policlinico.mi.it (A.B.); ludovica.leone.phd@gmail.com (L.L.); 2 Pediatric Intensive Care Unit, Foundation IRCCS Ca’ Granda Ospedale Maggiore Policlinico, 20122 Milan, Italy; 3 Department of Clinical Sciences and Community Health, University of Milan, 20122 Milan, Italy; martina.abodi@unimi.it; 4 Infertility Unit, Foundation IRCCS Ca’ Granda Ospedale Maggiore Policlinico, 20122 Milan, Italy

**Keywords:** children, adolescents, nutrition education, interactive learning, nutrition literacy, dietary behaviors

## Abstract

**Background**: Schools are widely recognized as key settings for promoting healthy eating behaviors and supporting childhood obesity prevention. In recent years, increasing attention has been devoted to experiential and interactive nutrition education strategies designed to actively engage children and adolescents in food-related learning processes. These approaches move beyond traditional didactic teaching and include practical and participatory formats, such as cooking activities, school gardening, digital or app-based learning tools, workshops and educational camps, and game-based learning interventions. **Objective**: This narrative review aims to provide an overview of experiential school-based nutrition education interventions, describing the main types of programs implemented in school settings and summarizing their reported effects on nutrition knowledge, attitudes, and eating behaviors among children and adolescents. **Results**: Across intervention studies and systematic reviews, hands-on and interactive educational models, including cooking classes, gardening programs, digital learning tools, workshops or camps, and board game-based interventions, frequently report improvements in nutrition knowledge, attitudes toward food, food-related skills, and self-efficacy. These programs seek to strengthen food literacy by combining experiential learning with educational content delivered within the school environment. Evidence regarding changes in dietary intake, diet quality, and anthropometric outcomes is more heterogeneous, with some studies reporting improvements in eating behaviors and others showing more modest or short-term effects. Program outcomes appear to be influenced by several contextual factors, including intervention duration, curriculum integration, teacher involvement, and the availability of resources supporting implementation. **Conclusions**: Experiential and interactive approaches represent an increasingly adopted strategy in school-based nutrition education. Their effectiveness appears to depend on the quality of implementation, the degree of integration within the school curriculum, and the broader educational context. Future research should further explore how different experiential formats can be optimally integrated into school systems to support the development of food literacy and sustainable healthy eating behaviors among children and adolescents.

## 1. Introduction

Schools are widely recognized as strategic settings for promoting healthy eating behaviors and supporting the prevention of childhood overweight and obesity, given their broad reach and sustained contact with children across formative developmental stages [1,2,3]. Consequently, school-based nutrition education has become a central component of public health strategies worldwide, implemented through curriculum-integrated lessons, experiential learning activities, multi-component programs involving families and communities, and broader policy and environmental changes within school systems [4].

A substantial body of literature suggests that these interventions frequently succeed in improving proximal outcomes, such as nutrition knowledge, attitudes, cooking skills, self-efficacy, and food-related preferences among children and adolescents. Innovative programs embedded within school curricula, experiential approaches, such as cooking and tasting activities, and multi-layered interventions engaging teachers, parents, and school environments, have demonstrated consistent gains in nutrition literacy and psychosocial determinants of dietary behavior. Moreover, integrative educational models designed to overcome curricular time constraints and sustainability-focused initiatives have gained increasing attention as potentially scalable solutions [5].

However, the translation of these cognitive and behavioral shifts into sustained improvements in dietary intake and obesity-related outcomes remains inconsistent. Several evaluations and systematic reviews report mixed or null effects on fruit and vegetable consumption, diet quality, and body mass index, despite high program acceptability and short-term improvements in knowledge or skills [6,7]. Implementation challenges, including limited classroom time, insufficient educational resources, reduced intervention intensity, and disruptions to program fidelity, emerge as recurrent barriers across educational contexts. Furthermore, methodological limitations and inadequate reporting of program content and delivery often restrict the ability to identify which components are truly effective.

Alongside these practical concerns, growing attention has been directed toward the potential unintended consequences of health promotion messaging in childhood. Simplistic narratives that frame foods as inherently “good” or “bad,” or that emphasize weight control as a primary goal, may inadvertently encourage dietary restriction, anxiety around eating, or stigmatizing attitudes toward body size, outcomes that conflict with long-term goals of healthy, autonomous food relationships. While many school-based programs aim to foster food literacy and empowerment, the broader educational discourse warrants critical examination to ensure that nutritional guidance supports both physical and psychosocial well-being.

Although numerous systematic and umbrella reviews have examined school-based nutrition interventions, the existing literature is largely structured along three main lines: broad evaluations of overall school-based programs, analyses focusing on specific outcomes or components (e.g., parental involvement, intervention intensity, or measurement tools), and, more recently, reviews targeting single delivery modalities, such as digital or media-based interventions. While these contributions have significantly advanced the field, they also focus on a specific modality (e.g., digital tools) and remain conceptually fragmented, with no examination within a unified framework. To date, no review has provided a comprehensive and integrative synthesis of these non-didactic educational modalities as distinct pedagogical strategies within school-based nutrition education.

This narrative review aims to address this gap by systematically examining and comparing innovative, non-traditional approaches to nutrition education across school settings.

## 2. Materials and Methods

This narrative review critically examines the literature on school-based nutrition education programs in children and adolescents, with a specific focus on intervention effectiveness, implementation barriers, and features associated with limited or inconsistent outcomes. A narrative approach was selected to allow for thematic integration of diverse study designs, intervention models, and contextual insights that extend beyond quantitative effectiveness estimates.

### 2.1. Literature Sources and Selection

A systematic literature search was conducted in February 2026 across three electronic databases: PubMed, Embase, and Web of Science. The search aimed to identify studies evaluating school-based nutrition education interventions delivered through interactive or experiential approaches, including game-based, digital, cooking, or gardening activities. More details about the search strategy can be found in Supplementary Materials. 

Studies were eligible for inclusion if they: (1) are quasi-experimental, intervention, randomized-controlled studies; (2) evaluated non-conventional nutrition education interventions delivered within school settings; (3) involved children or adolescents; and (4) reported outcomes related to nutrition knowledge, dietary behaviors, food literacy, psychosocial determinants (e.g., self-efficacy, preferences), or anthropometric indicators.

Articles were excluded if they: (1) included adult populations; (2) were not published in English; (3) consisted of review articles, editorials, study protocols or commentaries; (4) were not conducted in the school setting; (5) focused exclusively on traditional didactic or lecture-based nutrition education without interactive or experiential components; or (6) conducted in rural areas or low income populations; (7) included parents in the study population; (8) focused on pathological populations.

All study designs evaluating school-based interventions were considered eligible, including randomized controlled trials, cluster-randomized trials, quasi-experimental studies, and pilot intervention studies. Studies reporting both positive and mixed or null outcomes were included to allow a comprehensive appraisal of the evidence.

### 2.2. Data Extraction and Synthesis

Data were extracted from the included studies to summarize the main characteristics of the interventions. Studies were grouped according to the type of educational strategy used, including: (1) game-based nutrition education interventions, (2) web- and computer-based interventions, and (3) gardening- and cooking-based educational interventions.

Within each category, the results were qualitatively summarized by focusing on the main reported outcomes, including nutrition knowledge, dietary behaviors, food literacy, psychosocial determinants (e.g., self-efficacy, preferences, willingness to try foods), and anthropometric indicators when available. The synthesis aimed to highlight recurring patterns in the effectiveness of different intervention approaches and to identify similarities and differences in reported outcomes across studies.

Given the narrative and interpretative nature of this review, and the substantial heterogeneity in study designs, intervention modalities, and outcome measures, a formal risk-of-bias assessment using standardized tools was not conducted.

### 2.3. Data Synthesis

Rather than quantifying pooled effects, this review adopted a critical interpretative lens to explore why school-based nutrition education frequently underperforms in achieving sustained dietary change, despite improvements in knowledge and skills. Emphasis was placed on identifying recurring patterns of limited effectiveness, systemic constraints within school systems, and risks associated with overly simplistic or weight-centered health narratives.

## 3. Results

The database search identified a total of 4945 records across PubMed, Embase, and Web of Science. After removal of 2993 duplicate records, 1953 articles remained for title and abstract screening. During the initial screening phase, studies were excluded if they were out of scope, review articles, observational studies, focused primarily on physical activity interventions, not conducted in school settings, or conducted in adult populations. Only studies evaluating school-based nutrition education interventions delivered through non-lecture-based approaches were considered eligible for further assessment.

The remaining potentially relevant articles were subsequently assessed for inclusion in the narrative synthesis. Ultimately, 40 articles were included in the review.

### 3.1. Game-Based Nutrition Education Interventions

Several studies evaluated the effectiveness of game-based approaches in improving nutrition-related outcomes among children and adolescents in school settings. The interventions vary in format, including board games, digital games, and gamified educational platforms, but share a common educational and motivational approach. Study characteristics and results are shown in Table 1 and Table 2, respectively. Overall, these interventions showed consistent improvements in nutrition knowledge, attitudes, and psychosocial determinants of healthy eating, although evidence for sustained dietary behavior change was more heterogeneous.

A randomized controlled trial involving 50 primary school children aged 9–11 years evaluated the Food Hunter board game delivered weekly for four weeks. Food Hunter is a structured educational board game teaching food groups, healthy habits, and consequences of choices through gameplay [8]. Children showed significant gains in knowledge, attitudes, and self-efficacy (*p* < 0.001) and moderate behavioral improvements (*p* < 0.05) compared with the control group, with positive correlations observed between game engagement and improvements in both attitudes and behaviors.

Similarly, VitaVillage, a serious digital game set in a virtual village, where players complete quests related to food choices, cooking, and nutrition scenarios was tested among children aged 9–12 years. The study found high acceptability (>80% satisfaction) and trends toward improved knowledge after only two short gameplay sessions, though many results were not statistically significant (*p* > 0.05) due to small sample size. These findings support the potential feasibility of serious games as educational tools in school environments [9].

**Table 1:** Game-based studies characteristics.

Authors, Year	Country	Study Design	Study Duration	Intervention Type	Follow-Up	Population Age	Sample Size	Control Group YES/NO (If Yes, Details)
Çamlıbel et al., 2025 [8]	Turkey	Experimental-RCT (pre-test post-test)	4 weeks	The “FoodHunter” board game, designed to make learning about nutrition, engaging and interactive, thereby encouraging children to make healthier food choices.	NA	9–11 years	50	YES No intervention Intervention: *n* = 24 Control: *n* = 26
Chagas et al.,2020 [10]	Brazil	Cluster-RCT	7–17 days	Rango Cards: a game-based nutritional intervention on food consumption, nutritional knowledge and self-efficacy in the adoption of healthy eating practices	NA	13–19 years	319	YES Any intervention Intervention: *n* = 117 Control: *n* = 202
de Vlieger et al., 2021 [9]	Australia	Case-control study	2 occasions	VitaVillage: a farming-style game in which the player undertakes quests and completes questions aimed at increasing several aspects of nutrition and healthy eating knowledge	1 week	9–12 years	169	YES Intervention: *n* = 75 Control: *n* = 95
Hermans et al., 2018 [11]	Netherlands	Experimental design; 2 arms intervention study	2 days	The Alien Health Game: a video game designed to teach elementary school children about nutrition and healthy food choices.	2 weeks	10–13 years	108	YES A web-based nutrition game (active control condition) Intervention: *n* = 50 Control: *n* = 58
Joyner et al., 2017 [12]	USA	Case- control study	10–16 days	FIT GAME, a game in which children’s vegetable consumption influences events in a good versus evil narrative presented in comic book-formatted episodes.	Baseline I (no game): 10 days in both schools. FIT game I: 10 days in School I and 16 days in School P. Baseline II (no game): 6 days in School I and 4 days in School P. FIT game II: 6 days in both schools.	6–11 years	572	YES School P (episodes printed on posters) = 278 School I (episodes presented as images projected onto a screen) = 294
Salahshoornezhad et al., 2022 [13]	Iran	RCT	10 weeks	Smartphone game included into a multi-disciplinary intervention, (nutrition education using a smartphone game, aerobic exercise, and CBT)	NA	6–10 years	62 (overweight and obese)	YES Usual traditional nutritional education Intervention: *n* = 31 Control: *n* = 31
Sharma et al., 2015 [14]	USA	Quasi-experimental group-RCT	6 weeks	QTLM, an immersive three-dimensional action-adventure game on dietary behaviors, physical activity behaviors, and psychosocial factors; recommended game exposure duration was 90 min/week.	NA	9–10 years	94	YES No intervention (usual school program) Intervention: *n* = 44 Control: *n* = 50
Stival et al., 2026 [15]	Italy	Uncontrolled intervention study	7 months	Food Game, a secondary school-based program to promote healthy eating, physical activity, and sustainability awareness	NA	14–16 years	184	NO
Viggiano et al., 2015 [16]	Italy	Cluster-RCT	20 weeks	Kaledo, a new board game, promotes nutrition education and improves dietary behavior.	6 months And 18 months	9–19 years	3110	YES Any intervention Intervention: *n* = 1663 Control: *n* = 1447
Wang et al., 2025 [17]	China	Pilot Study	2 weeks	HFHM an alternate reality game to enhance their nutrition knowledge and improve their eating behaviors	NA	7–8 years	79	YES No game Intervention: *n* = 40 Control: *n* = 39

Abbreviations: CBT: Cognitive Behavioral Therapy; HFHM: Happy Farm, Happy Meal; NA: not applicable; QTLM: Quest to Lava Mountain.

**Table 2:** Game-based studies: results and conclusions.

	Outcomes Results			
Authors, Year	Nutrition Knowledge	Anthropometric Measurements	Food Choices/ Dietary Intake	Conclusions
Çamlıbel et al., 2025 [8]	The FBS, CDSS, and NAS scores increased statistically significantly in the intervention group post intervention (*p* < 0.05); the FBS score decreased significantly in the control group (*p* < 0.05).	NA	Consumption of breakfast, snacks, milk-yogurt, cheese, egg, vegetables, and fruit in the intervention group increased significantly; packaged products, cake-pastries, and sugary beverages decreased significantly (*p* < 0.05). In the control group, consumption of cheese, fruit, and bread decreased, while the consumption of cakes-pies-cookies, sugary drinks, ready meals, and sausage-salami increased within group (*p* < 0.05).	This study confirms the effectiveness of the developed FoodHunter game intervention in improving primary school children’s nutritional behaviors, self-efficacy, and attitude.
Chagas et al., 2020 [10]	The intervention group showed increased knowledge of the effects of fruit and vegetable consumption (*p* = 0.033), improved self-efficacy in the adoption of healthy eating practices, such as reducing salt intake (*p* = 0.032) and preparing healthy meals (*p* = 0.031)	NA	Significant decrease in eating while watching TV or studying and having meals at fast food restaurants in the intervention group (*p* = 0.042)	This study found an impact of the intervention on the habit of eating while watching TV or studying; on having meals at fast food restaurants; on knowledge related to the importance of fruit and vegetable consumption and on self-efficacy in the adoption of healthy eating practices, such as reducing Na intake and preparing healthy meals.
de Vlieger et al., 2021 [9]	Nutrition knowledge increased by 2.25 points (mean) in the intervention group between T1 and T2, (SD) 6.31, *p* = 0.035) compared to controls	NA	Moderate changes in nutrition knowledge scores from T1 to T2 between control and intervention for the categories “AGHE and Balanced meals”. The latter was significant (*p* = 0.006).	Overall nutrition knowledge in the intervention group increased after playing Vita Village; mainly for the categories ‘AGHE serves’, ‘Food categorisations’, ‘Nutrition labels’ and ‘Balanced meals’. Preliminary data shows some improvement in nutrition knowledge after playing VitaVillage for a short time. Long term results are to be tested.
Hermans et al., 2018 [11]	Children who played the game increased knowledge at immediate post-test, *t* (105) = 4.45, *p* < 0.001, but not at 2-week-follow-up, (*t* (105) = 1.30, *p* = 0.999).	NA	Participants were better able to select the healthier food item out of two options over time, but this effect did not differ for those in the experimental versus the control condition.	Children in the experimental condition showed better immediate recall of the main function of macronutrients than those in the control condition; strong retention was not seen in the 2-week delayed test. Furthermore, no differences were found in children’s knowledge of the function of the five most important macronutrients. Two short bouts of Alien Health can increase some types of knowledge in the short term, but may not be strong enough to significantly increase children’s nutritional knowledge and actual eating behavior in the long term.
Joyner et al., 2017 [12]	NA	NA	During Phase I, vegetable consumption increased by 69% to an average of 36.8 g per child per day (School P: *R* = 0.61, *p* = 0.05, *d_av_ =* 0.74; School I: *R* = 0.34, *p* < 0.05, *d_av_ =* 0.76); n Phase II, vegetable consumption increased from Baseline II by 181% to an average of 57.5 g per child per day (School P: *R* = 0.98, *p* = 0.0001, *d_av_ =* 8.84; School I: *R* = 0.81, *p* = 0.03, *d_av_ =* 2.44).	FIT Game is a low-cost, low-labor intervention that can positively impact healthy eating in elementary schools, although its impact is limited to times when the game is played.
Salahshoornezhad et al., 2022 [13]	NA	In the intervention group, a significant decrease was seen in weight (*p* < 0.001), BMI (*p* < 0.001), and HC (*p* < 0.001) compared to the beginning measurement. Weight (*p* < 0.001), height (*p* < 0.001), BMI (*p* = 0.01), waist (*p* < 0.001), and HC (*p* = 0.001) changes were significant at the end of the study when compared between groups.	Eating habits, considering DEBQ scores: emotional (*p* < 0.001) and external *p* < 0.001) changes decreased, and dietary restraint (*p* < 0.001) increased significantly in both groups, but these changes were more significant in the intervention group.	Results showed that a 10-week smartphone game-based intervention with CBT and regular physical activity three times a week had a better effect on weight loss, WC and HC, and appetite score compared to the control group. The results indicated that the training provided to children through smartphone games could affect their performance in real life and be more attractive to children.
Sharma et al., 2015 [14]	Significant increase in nutrition and physical activity-related attitudes (adjusted β = 0.85, 95% CI 0.24–1.45; *p* = 0.006); decrease in nutrition/physical activity knowledge (*p* = 0.038).	NR	Significant decreases pre-intervention to post-intervention in the amount of sugar among children (−4.9 g/1000 kcal in the intervention group vs. +5.61 g/1000 kcal in the comparison group; *p* = 0.021). The decreases in the comparison group were greater than the intervention group for energy intake (−111 kcal in the intervention group vs. −302 kcal in the comparison group; *p* = 0.003).	The QTLM computer-based education game has promising acceptability and initial effects on significantly decreasing intake of sugars and improving nutrition, physical activity attitudes, and behaviors among ethnically diverse elementary school aged children. Children perceived QTLM as a useful and enjoyable avenue to encourage healthy food choices.
Stival et al., 2026 [15]	Waste recycling remained consistently high (95%), and the use of tap water showed a slight, non-significant increase.	NA	Students reported a higher frequency of fruit (*p* = 0.014), vegetable (*p* = 0.002), and fish consumption (*p* = 0.013) after the program, while intake of meat (*p* = 0.004), processed meat (*p* = 0.004), and snacks (*p* = 0.007) decreased significantly. 29.4% of students achieved a substantial improvement in dietary score (≥2-point increase).	The gamification-based Food Game program is promising for promoting adolescents’ healthy behaviors. These improvements, even if modest, may represent early behavioral shifts with the potential to consolidate into long-term dietary habits.
Viggiano et al., 2015 [16]	6 months post-treatment assessment: a significant difference in the mean values adjusted for the score at baseline between the treated group and the control group (6.5 vs. 4.6 *p* < 0.001) for nutrition knowledge. No significant differences at 18 months post-intervention.	The treated group significantly descreased BMI z-score with respect to the controls at 6 (0.44 vs. 0.58), *p* = 0.001) and 18 months (0.34 vs. 0.58 *p* = 0.017) post-assessments. % normal, overweight, and obese subjects in the treated group were significantly different at both 6 (*p* = 0.038) and at 18 months *p* = 0.004) compared with baseline.	At 6 months post-treatment assessment: a significant difference in the mean values adjusted for the score at baseline between the treated group and the control group (11.2 vs. 10.4 *p* < 0.001) for healthy vs. unhealthy diet and foods, and for “food habits” (32.4 vs. 27.64 (27.3 to 28.0); *p* < 0.001) No significant differences at 18 months post-intervention.	Kaledo improved nutrition knowledge and dietary behavior over 6 months and had a sustained effect on the BMI z-score at 6 and 18 months after the game.
Wang et al., 2025 [17]	The HFHM group showed a significant increase in nutrition knowledge (*p* < 0.05).	NA	The HFHM group showed significantly reduced food waste (*p* < 0.01), decreased picky eating (*p* < 0.01), and improved meal duration (*p* < 0.05).	HFHM is a promising tool for improving nutrition education and dietary behaviors in Chinese children.

Abbreviations: BMI: body mass index; CBT: Cognitive Behavioral Therapy; CDSS: Children’s Dietary Self-Efficacy Scale; DEBQ: Dutch Eating Behavior Questionnaire; FBS: Food Behavior Scale; HC: hip circumference; NA: not applicable; NAS: Nutrition Attitude Subscale; NR: not reported.

In adolescents, the cluster-randomized trial evaluating the Rango Cards, a digital card-based educational game where players categorize foods and build balanced meals, promoting discussion and decision-making, was tested among 319 high school students. The intervention improved nutrition knowledge (*p* < 0.001) and self-efficacy (*p* < 0.01), with moderate dietary improvements (*p* < 0.05), particularly increased fruit consumption, together with reductions in some unhealthy eating practices, such as eating while watching television or studying and frequent fast-food consumption [10]. These findings suggest that game-based approaches may promote greater autonomy and self-management in dietary choices during adolescence.

Other interventions produced more modest or short-term effects. The Alien Health Game, a digital game where children “feed” an alien by selecting foods, learning which are healthy or unhealthy through feedback was tested among Dutch children aged 10–13 years. Participants showed higher nutrition knowledge of macronutrients (*p* < 0.001) and increased selection of healthy snacks (*p* < 0.05), immediately after gameplay, although these effects were not sustained at follow-up and no significant changes were observed in actual food intake [11].

Conversely, some interventions targeting school environments demonstrated measurable changes in dietary behavior. The FIT Game, implemented in elementary school cafeterias and involving over 500 students, was a cafeteria-based gamified system where students collectively achieve goals (e.g., eating vegetables) to advance a story and earn rewards [12].

Vegetable consumption increased by ~20–30% (*p* < 0.01), compared with baseline levels through a narrative-driven game mechanism linking students’ vegetable intake to the progression of a story [12]. The study highlighted reduced implementation costs while maintaining effectiveness.

Another pilot cluster-randomized trial evaluated the Quest to Lava Mountain among ethnically diverse elementary school children [14]. This computer-based adventure game where children complete challenges by making healthy diet and physical activity choices to progress through levels, reported reduced sugar consumption and improved nutrition attitudes, such as small but significant increases in fruit/vegetable intake (about +0.3–0.5 servings/day, *p* < 0.05), although effects on physical activity and other dietary outcomes were limited. The game was engaging, though limited by short exposure and pilot design.

Kaledo, a board game simulating daily life decisions (meals, shopping, activity), where players accumulate or lose points based on healthy behaviors, was evaluated in a large cluster-randomized trial, including more than 3000 students aged 9–19 years [16].

Children showed significant improvements in healthy eating behaviors (*p* < 0.05), nutrition knowledge (*p* < 0.001), and lifestyle indicators, together with a significant reduction in BMI z-score at both 6 (*p* < 0.001) and 18-month (*p* = 0.017) follow-up, suggesting potential longer-term benefits of repeated game-based education sessions [16]. Moreover, the percentage of normal, overweight, and obese subjects in the treated group was significantly different at both 6 (*p* = 0.038) and 18 months (*p* = 0.004) compared with baseline.

Taking into account anthropometric measurements, similar results were found by Salahshoornezhad et al. [13]. The intervention was not purely a game: it embedded gamification elements within a broader multidisciplinary program, including diet, physical activity, cognitive behavioral therapy (CBT) and clinical monitoring, in a cohort of overweight and obese girls. Significant improvements were seen in BMI (−1.5 to −2.0 kg/m^2^, *p* < 0.01) and biochemical markers, such as LDL cholesterol (*p* < 0.05), showing stronger outcomes than game-only interventions.

Board-game studies also investigated another important topic, that is sustainability. The pilot study Happy Farm, Happy Meal (HFHM) by Wang et al., conducted with Chinese children, was an alternate reality game integrating real-world and digital tasks, where children solve missions and challenges related to food and health in everyday environments, to enhance their nutrition knowledge and improve their eating behavior [17]. The study reported significant increases in knowledge (*p* < 0.001) and self-reported healthy behaviors (*p* < 0.05), and significantly reduced food waste (*p* < 0.01). Similarly, a recent Italian intervention study [15], tested the Food Game, a gamified school-based program combining quizzes, challenges, and sustainability-themed tasks focused on diet and lifestyle. Results showed greater sustainability awareness, such as the use of tap water and waste recycling, although not statistically significant, and improvements in healthy food consumption (*p* < 0.01) and reduced ultra-processed food consumption (*p* < 0.05) after the program.

Overall, these findings suggest that game-based nutrition education is a promising strategy to enhance engagement and improve knowledge, attitudes, and some dietary behaviors among children and adolescents. However, the magnitude and persistence of behavioral changes vary across studies, likely depending on intervention intensity, duration, and integration within the school environment.

The extent of these changes is generally small, and in several cases (e.g., The Alien Health Game, VitaVillage), improvements in actual intake are limited or inconsistent despite strong gains in knowledge, suggesting a gap between learning and sustained behavior change. Additionally, heterogeneity in intervention formats and outcome measures limits comparability across studies, and few interventions assess objective health outcomes [13].

### 3.2. Web- and Computer-Based Interventions

Across the reviewed literature, digital and web-based nutrition interventions in adolescents vary substantially in structure and effectiveness, but some consistent patterns emerge when examining their specific features and outcomes. Study characteristics and results are shown in Table 3 and Table 4, respectively. The interactive, web-based, tailored school program platform proposed in the study conducted by Chamberland et al. [18], delivered personalized feedback, goal setting, quizzes, and tracking tools targeting fruit/vegetable and milk intake. Results showed significant increases in fruit and vegetable intake (~+0.3 servings/day, *p* < 0.05) and milk/alternatives consumption (*p* < 0.05), although no effects on BMI or total energy intake were observed (*p* > 0.05). Moreover, outcomes were stronger among more engaged users. This study represents a structured, behavior-change-oriented web intervention.

Similarly, the StayingFit Brazil program [19], a web-based/computer-delivered educational program with modules, games, quizzes, and behavior-change techniques (multi-component digital curriculum), achieved significance in dietary behaviors in the intervention group, such as the reduction (35%) of soft drinks consumption (OR = 0.65; 95% CI 0.50–0.84) and a greater (43%) consumption of beans (OR = 1.43; 95% CI 1.10–1.86). The intervention had no effect on anthropometric and body composition measurements (*p* > 0.05).

In contrast, the HELENA Food-O-Meter [20], a computer-tailored web tool generating automated personalized feedback by comparing self-reported intake with dietary guidelines, improved knowledge and attitudes (*p* < 0.01) but led to inconsistent and non-significant dietary changes (*p* > 0.05), suggesting that personalization without sustained engagement or behavioral support may be insufficient.

A different and notably more effective approach is represented by intervention implemented directly in school canteen/cafeterias aimed at improving food choices firstly at school lunch. The Click and Crunch High Schools [21] program, web-based choice architecture strategies—such as default healthy options, traffic-light labeling, and strategic item positioning—were embedded directly into online school canteen ordering systems. This was the environmental digital intervention that produced the strongest behavioral outcomes across studies, significantly improving the nutritional quality of purchases and increasing selection of healthier items (*p* < 0.001), without requiring active educational engagement.

**Table 3:** Web-based studies characteristics.

Authors, Year	Country	Study Design	Study Duration	Intervention Type	Follow-Up	Population Age	Sample Size	Control Group YES/NO (If Yes, Details)
Bordeleau et al., 2023 [22]	Canada	Cluster- RCT	6 weeks	A web-based school nutrition intervention; a nutrition challenge using a web-based platform—Team Nutriathlon aimed at improving diet quality by increasing and adding diversity to their consumption of vegetables/fruits and dairy products.	Three follow-ups, each one every two weeks.	13 years	237	YES Regular school curriculum Intervention: *n* = 162 Control group: *n* = 75
Brito Beck da Silva et al., 2019 [19]	Brazil	Cluster- RCT	12 months	StayingFit is an online program organized to encourage and guide weight control and healthy eating habits based on the tenants of CBT.	NA	12–14 years	895	YES (details not reported) Intervention: *n* = 428 Control group: *n* = 467
Chamberland et al., 2017 [18]	Canada	Cluster-RCT	6 weeks	Team Nutriathlon, an innovative web-based platform to promote the consumption of vegetables and fruit and milk and alternatives in high school students.	10 weeks after no intervention	12–13 years	282	YES Regular school curriculum Intervention: *n* = 193 Control: *n* = 89
Delaney et al., 2022 [21]	Australia	Cluster- RCT	2 months	“Click and Crunch High Schools”, a 2-month choice architecture intervention (involving menu labeling, prompts, item positioning, and feedback)	2 months after the 2-months intervention.	14–18 years	1331	YES Usually online ordering, no change to their online canteen menu Intervention: *n* = 656 Control group: *n* = 675
Fassnacht et al., 2014 [23]	Portugal	Case-control pilot study	8 weeks	Mobile phone SMS to promote healthy behaviors.	4 weeks after no intervention	8–10 years	49	YES No-monitoring group Intervention: *n* = 22 Control: *n* = 27
Gilliland et al., 2026 [24]	Canada	Pilot study	8 weeks	SmartAPPetite application on phone, which sent time-based healthy eating messages (max 3/day) and location-based “nudge” messages (max 5/day)	NA	14–17 years	54	NO
Long et al., 2004 [25]	USA	Quasi-experimental	1 month	A combination of 5 h of web-based instruction and 10 h of classroom curriculum.	NA	12–16 years	121	YES Nutrition education embedded in the standard school curriculum (Intervention *n* = 63; Control: *n* = 58)
Maes et al., 2011 [20]	Six European Cities	Quasi-experimental	3 months	Computer-tailored advice	1 month and 3 months	12–17 years	1298	YES Standardized advice (Intervention: *n* = 713; Control: *n* = 585)
Turnin et al., 2016 [26]	France	Quasi-experimental	1 year	Nutri-Advice software, available on multimedia kiosks consisting of a computer, a touch screen, and a bar code scanner.	NA	12–14 years	580	NO

Abbreviations: CBT: Cognitive Behavioral Therapy; NA: not applicable; SMS: short message service.

**Table 4:** Web-based studies: results and conclusions.

	Outcomes Results			
Authors, Year	Nutrition Knowledge	Anthropometric Measurements	Food Choices/ Dietary Intake	Conclusions
Bordeleau et al., 2023 [22]	NR	No differences in anthropometric measurements.	Post hoc analyses revealed a significant reduction in susceptibility to hunger among boys in the intervention group for pre-intervention and post-intervention (*p* = 0.01). Some eating behaviors improved, but not all reached statistical significance.	The school-based nutrition intervention challenge did not negatively impact eating behavior traits, body weight concern, body size perception and dissatisfaction in adolescents; moreover, this type of intervention could reduce susceptibility to hunger among adolescent boys.
Brito Beck da Silva et al., 2019 [19]	NA	No differences in the observed anthropometric parameters were observed (such as BMI, body composition, waist circumference).	Significant reduction (35%) in soft drinks consumption (OR = 0.65; 95% CI 0.50–0.84) and greater (43%) consumption of beans (OR = 1.43; 95% CI 1.10–1.86) in the control group.	The results of the present study suggest a positive effect of the adapted version of StayingFit with regard to an increase in the frequency of beans consumption and a decrease in the frequency of soft drink consumption among elementary school students. It appears that implementing a web-based intervention would be beneficial and could be used to promote changes in eating habits and health among adolescents.
Chamberland et al., 2017 [18]	NA	NA	Significant increase of three servings per day of fruit and vegetables, and 1.8 servings per day of milk and alternatives in the intervention group (*p* < 0.05).	Team Nutriathlon represents an innovative web-based nutrition program, which positively impacts fruit and vegetables as well as milk and alternatives consumption among high school students.
Delaney et al., 2022 [21]	Significant between group differences over time favoring the intervention group for the mean percentage of all online lunch items per student that were ‘Everyday’ (+5.5%; *p* < 0.001) and ‘Should not be sold’ (−4.4%; *p* < 0.001).	NA	No differences between group over time in the average energy, saturated fat, sugar, and sodium content of student online lunch orders.	A low intensity, choice architecture intervention embedded within an online ordering system can increase the purchase of healthier food items for high school students.
Fassnacht et al., 2014 [23]	NA	NR	Significant increase in fruit/vegetable intake in the intervention group (covariance analysis: χ^2^ [2] = 7.27; *p* < 0.05).	The results indicate that participation in the SMS program may be associated with positive behavioral changes. Participants in the intervention group consumed significantly more fruits and vegetables over time compared with the control group.
Gilliland et al., 2026 [24]	The app helped participants’ experience an increase in intake of local foods and gain more awareness about healthy recipes. 49.1% of students noted that the app helped improve their knowledge about food and nutrition, they feel eating healthily is more important to them and they liked to cook more after participating in SmartAPPetite.	NR	Changes in breakfast eating habits, while snacks, lunch, and dinner habits appeared to remain stable. Water consumption increased from 2.7 to 2.9 instances per day, while decreasing the consumption of energy-dense nutrition-poor foods, sports drinks and candy/chocolate per day.	The results of this pilot study show that SmartAPPetite is feasible, adolescents will accept the intervention and enjoy engaging with the app. Participants perceived that it had the potential to influence food knowledge, food purchasing, and food intake behaviors. Even if these are only preliminary results, this study demonstrates that the app has the potential to reach many adolescents within and beyond Southwestern Ontario.
Long et al., 2004 [25]	The intervention group had significantly higher scores for self-efficacy for fruits and vegetables (*p* < 0.01), self-efficacy for lower fat, (*p* < 0.001) usual food choices (*p* < 0.001), and dietary knowledge of fat (*p* < 0.05) compared to the control group.	NA	No significant changes	The intervention for increasing self-efficacy for healthy eating was effective, and such innovative methods of delivering nutrition education might help adolescents to lead healthier, longer lives.
Maes et al., 2011 [20]	NA	NA	At 3 months: overall fat intake decreased (F = 2·80, *p* < 0.10), especially in the intervention group, in overweight adolescents (F = 5·76, *p* < 0.05).	The implementation of a web-based tailored intervention was feasible and although generally well appreciated by the adolescents, the results were modest but clear for percentage energy from fat, specifically in the overweight group.
Turnin et al., 2016 [26]	NA	The three schools enrolled presented different results: Overall, BMI z-score and obesity percentage decrease significantly post-intervention (*p* < 0.001 and *p* < 0.05, respectively)	The three schools enrolled presented different results: Overall, significant increase in starch (*p* < 0.05) and fruit and vegetables consumption (*p* = 0.05), and significant reduction in dairy products (*p* < 0.05) and cheese (*p* < 0.01) and pastry, ice cream and desserts consumption (*p* < 0.001).	Children chose significantly less cheese and pastry or desserts, and significantly more starchy food and dairy, and tended to choose fruits and vegetables more often.

Abbreviations: BMI: body mass index; CI: confidential interval; OR: odds ratio; NA: not applicable; NR: not reported.

By comparison, Bordeleau and colleagues implemented a web-based psychoeducational platform called Team Nutriathlon, combining nutrition content with body image components (videos, reflective exercises, self-assessment), focused on eating behaviors and body size preoccupation in adolescents, which significantly enhanced intuitive eating, especially among boys (*p* < 0.01) and reduced body dissatisfaction but showed limited or non-significant effects on dietary intake and no anthropometric changes, highlighting a focus on psychological rather than behavioral outcomes [22].

Other interventions emphasize contextual or mobile delivery. For instance, Nutri-Advice [26] used interactive educational software in school cafeterias, that provided real-time feedback and suggestions, such as nutrition messages, recipes, and local food info to adolescents at the point of meal selection. During the intervention period there was a significant improvement in healthier food selections, such as more fruit, vegetables consumption and reduced high fat/sweet products, and improved nutritional knowledge (*p* < 0.01). Overall, BMI decreased significantly during the study period (*p* < 0.001), and obesity percentage decreased significantly (*p* < 0.05) in one of the three schools enrolled.

The SmartAPPetite for Youth study [24,27], a smartphone-based app offering push notifications, local healthy food suggestions, recipes, and goal-setting features, was conducted among adolescents. It demonstrated high feasibility and acceptability (>75–80% satisfaction/retention) but only non-significant trends in dietary improvement due to its pilot nature. Nevertheless, the consumption of healthy and unhealthy foods of participants showed positive trends in the pre-post comparison, suggesting SmartAPPetite has the potential to positively impact food intake. By contrast, participants less frequently consumed sports drinks and candy/chocolate per day.

Likewise, Fassnacht et al. showed that a low-cost SMS-based intervention delivering regular prompts and motivational messages could produce modest but significant improvements in some health and dietary behaviours, such as the increased consumption of fruit and vegetables (*p* < 0.05), illustrating the potential of simple, scalable push strategies [23].

Finally, Long & Stevens provided early evidence that computer-based, theory-driven interventions using interactive scenarios and feedback significantly improve self-efficacy for healthy eating and nutritional knowledge in the intervention group (*p* < 0.01), although dietary changes remained non-significant [25].

Taken together, these studies illustrate that technology-based nutrition interventions for adolescents can leverage multiple digital platforms, websites, computer software, mobile apps, SMS services, and online decision systems, to improve dietary behaviors. While most interventions demonstrated beneficial effects on self-reported eating habits or food choices, changes in anthropometric measures, such as BMI, were generally absent or minimal within the short to medium intervention durations studied. Interventions that combined self-monitoring, personalized feedback, environmental nudges, and behavioral prompts tended to produce stronger changes in dietary behaviors than those relying on education alone. However, most positive findings were based on self-report or purchase data, and longer-term follow-up is needed to determine whether such technology-enabled changes are sustained and translate into improvements in body composition or chronic disease risk.

### 3.3. Gardening and Cooking-Based Educational Interventions

The included studies indicate that experiential school-based interventions (gardening, cooking, and integrated gardening–cooking–nutrition education programs) have been implemented mainly in primary schools (around 7–12 years), with some after-school programs and fewer interventions in secondary schools. Study characteristics and results are shown in Table 5 and Table 6, respectively Overall, findings are more consistent for nutrition knowledge/food literacy and psychosocial determinants, whereas effects on dietary intake and diet quality (particularly sustained increases in vegetable intake) and on anthropometric indicators are more heterogeneous and appear to depend on program intensity, duration, and implementation quality.

Most programs were delivered in primary school children: second grade (≈7–8 years) in school-based cooking curriculum assessing attitudes and behaviors [28] and in a randomized controlled trial evaluating school gardens as an experiential nutrition education approach that improved fruit and vegetable knowledge, preference, and consumption among second-grade students [29]; fourth grade (≈9–10 years) in cooking/tasting curricula [21] and hands-on cooking programs [30]; 8–9 years in a quasi-experiment comparing practical cooking vs. nutrition education [30]; and 11–12 years in garden-plus-curriculum interventions [31] or integrated garden-based programs, including education and cooking [32,33]. After-school interventions included LA Sprouts (grades 3–5, ≈8–11 years) [34] and the extracurricular culinary program Vetri Cooking Lab (grades 3–11) [35]. A curriculum-based secondary school intervention (optional 18-week culinary course) was also reported [36].

Among integrated gardening–cooking–nutrition interventions, the cluster-randomized trial TX Sprouts (primary schools; predominantly low-income population with a high proportion of Hispanic children) showed measurable effects on diet quality and dietary composition. Compared with controls, the intervention improved the HEI-2015 “Total Vegetables” component (+4% vs. −2%; *p* = 0.003) and modestly increased the percentage of energy from protein (+0.4% vs. −0.3%; *p* = 0.021). In addition, the increase in added sugars was smaller in the intervention group (0.3 vs. 2.6 g/day; *p* = 0.050) [37]. A subsequent NOVA-based analysis reported higher consumption of unprocessed/minimally processed foods (+2.3% vs. −1.8%; *p* < 0.01) and lower ultra-processed foods (UPF) (−2.4% vs. +1.4%; *p* = 0.04) in the intervention group [38]. Anthropometric outcomes were not reported in these TX Sprouts contributions.

In an analysis linking changes in cooking/gardening behaviors to outcomes, increases in these behaviors were associated with dietary improvements: higher fiber intake (cooking *p* = 0.004; gardening *p* = 0.02) and higher vegetable intake associated with cooking behaviors (*p* = 0.03), with no association with BMI z-score or waist circumference [39].

Across garden-based interventions, evidence for increasing total fruit and vegetable intake was mixed. A large UK cluster-RCT (mean age 8.1 years; 18-month follow-up) did not show a statistically significant adjusted overall intervention effect (−40 g; 95% CI −88, 1; *p* = 0.06). However, process measures indicated that, irrespective of group allocation, a three-level increase in school gardening implementation was associated with a mean increase of +81 g/day in fruit and vegetable intake (95% CI 0, 163; *p* = 0.05) [40]. In Jordan (10–12 years; 5 months; garden + education + tasting), the intervention improved diet quality (fiber +2.36 g/day; saturated fat −9.24 g/day) and anthropometrics, with reductions in BMI (−1.57 kg/m^2^), weight (−1.88 kg), and BMI z-score (−0.37) compared with controls [33].

**Table 5:** Cooking and gardening studies characteristics.

Authors, Year	Country	Study Design	Study Duration	Intervention Type	Follow-Up	Population Age	Sample Size	Control Group YES/NO (If Yes, Details)
Caraher et al., 2013 [41]	UK	Quasi-experimental intervention study	1 year	A school cooking and nutrition intervention (“Chefs Adopt a School” programme) including healthy eating, tasting foods, food preparation, hygiene, and practical cooking skills	Pre-post intervention	9–11 years	162	YES No intervention during the study period. Control group: *n* = 81
Christian et al., 2014 [40]	UK	Cluster-RCT	6–12 months	School gardening intervention (Royal Horticultural Society “Campaign for School Gardening”)	Pre-post intervention	8–10 years	641	YES Schools continuing usual curriculum without structured gardening intervention
Cunningham-Sabo et al., 2014 [28]	USA	Non-randomized-interventional study	10 weeks	School-based nutrition education program, including hands-on cooking and tasting activities	Pre-post intervention	9–10 years	231	YES Control classes continued with the standard school curriculum
Davis et al., 2015 [42]	USA	Cluster-RCT	12 weeks	Multi-component program (gardening + nutrition education + cooking)	Pre-post intervention	9–11 years	364	YES Usual after-school program without gardening/cooking/nutrition curriculum
Elsahoryi et al., 2025 [33]	USA	Quasi-experimental controlled trial	5 months	Gardening intervention	Pre-post intervention	10–12 years	216	YES control group (*n* = 95)
Jaenke et al., 2012 [31]	Australia	Cluster-controlled	NA	Multi-component: Nutrition education ± school garden + cooking program	Pre–post intervention	10–12 years	127	YES Standard school curriculum (no gardening/cooking intervention)
Jeans et al., 2023 [38]	USA	RCT	3 years	Multi-component (gardening + nutrition education + cooking)	NA	9 years	451	YES control group: *n* = 228
Kearney et al., 2024 [35]	UK	Longitudinal observational study	1 year	Multi-component (nutrition education + cooking)	No	8 years	171	NO
Kim et al., 2020 [32]	South Korea	Quasi-experimental	12 weeks	Multi-component (gardening + nutrition education + cooking)	Pre-post intervention	11–12 years	202	NO
Labbe et al., 2023 [43]	Australia	Quasi-experimental	3 months	Multi-component (nutrition education + cooking)	NA	10–11 years	170	YES control group: *n* = 82
Landry et al., 2019 [39]	USA	RCT	12 weeks	Multi-component (cooking + gardening program—LA Sprouts)	Baseline and immediate post-intervention	9 years	290	YES Usual after-school program (no structured cooking/gardening intervention)
Landry et al., 2021 [37]	USA	RCT	3 years		Follow-up	9.8 years	3302	YES. control group: *n* = 1723
LeBlanc et al., 2022 [36]	USA	Quasi-experimental	5 months	Cooking	NA	7 years	326	YES control group: *n* = 202
Maiz et al., 2021 [44]	Spain	Quasi-experimental	4 week	Multi-component (nutrition education + cooking)	Post-intervention	8–9	202	99 in the nutrition education group and 103 in the hands-on group.
Parmer SM et al., 2009 [29]	USA	RCT	4–5 months	School garden-based nutrition education (experiential learning)	Baseline and post-intervention reported)	7–8 years	115–120	YES Standard curriculum without gardening intervention
Zahr et al., 2017 [30]	Canada	Quasi-experimental	7–10 weeks	Cooking course (hands-on food preparation + education)	Pre- and post-intervention (no long-term follow-up)	10–12 years	90–100	NO

Abbreviations. NA: not applicable; RCT: randomized controlled trial.

**Table 6:** Cooking and gardening studies: results and conclusions.

	Outcomes Results			
Authors, Year	Nutrition Knowledge	Anthropometric Measurements	Food Choices/ Dietary Intake	Conclusions
Caraher et al., 2013 [41]	Children in the intervention group showed improvements in food and nutrition knowledge and cooking-related understanding compared with controls. Cooking confidence score increased from 3.09 to 3.35 in the intervention group (*p* < 0.001) and willingness to try healthier foods improved in the intervention group	NA	Vegetable consumption score increased from 2.24 to 2.46 (+0.22 points) after the intervention (*p* = 0.002, CI 0.01–0.18)	The chef-led cooking intervention improved children’s cooking confidence, engagement with healthy foods, and veg table consumption; school cooking programmes can positively influence children’s food behaviours, but consistent nutrition messaging and proper evaluation are important.
Christian et al., 2014 [40]	Not a primary outcome; limited or no significant effect reported	No (not a primary endpoint)	There was no significant difference in fruit and vegetable intake between intervention and control groups. At follow-up, mean daily fruit and vegetable intake did not differ significantly between groups (adjusted difference ≈ 0.08 portions/day, 95% CI −0.12 to 0.28; *p* > 0.05). Similarly, no significant effects were observed for fruit intake or vegetable intake analyzed separately (all 95% CIs included the null value and *p* > 0.05).	The school gardening intervention did not significantly improve children’s fruit and vegetable intake compared with controls, with only small, non-significant differences observed between groups. Findings should be interpreted with caution despite the randomized design, due to potential biases, including variability in intervention implementation, reliance on self-reported dietary intake, and possible limited intervention intensity.
Cunningham-Sabo et al., 2014 [28]	Students in the intervention group showed greater improvements in nutrition knowledge compared with controls (*p* < 0.001). The effect was more pronounced among students with lower prior cooking experience.	Anthropometric outcomes were not assessed.	Students in the intervention group showed improved fruit and vegetable preferences and willingness to try new foods (*p* < 0.05), with stronger effects in girls and those with lower prior cooking experience (interaction *p* < 0.05). No significant changes were observed in actual dietary intake (all *p* > 0.05).	The school-based cooking intervention improved nutrition knowledge and food-related attitudes, particularly among girls and students with lower prior cooking experience, but did not lead to changes in actual dietary intake; findings should be interpreted with caution due to the non-randomized design, self-reported outcomes, and short-term follow-up.
Davis et al., 2015 [42]	Students in the intervention group showed greater improvements in nutrition and gardening knowledge compared with controls (+14.5% vs. −5.0%; *p* = 0.003). They also demonstrated greater improvement in vegetable identification (+11% vs. +5%; *p* = 0.001).	Following the 12-week intervention, showed a modest reduction in BMI compared with controls (*p* < 0.05)	A higher proportion of students in the intervention group reported gardening at home compared with controls (+7.5% vs. −4.4%; *p* = 0.003). No significant baseline differences were observed between groups in determinants of dietary behavior, and groups remained comparable across demographic variables (all *p* > 0.05).	The intervention improved nutrition knowledge and behavioral determinants (e.g., vegetable identification, gardening behaviors), but interpretation should consider potential biases related to small cluster number, self-reported outcomes, and attrition.
Elsahoryi et al., 2025 [33]	Increased substantially in the intervention group (+22.31 points) compared to controls (+1.75 points; *p*-value ≤ 0.001).	Reductions in BMI (−1.57 kg/m^2^), weight (−1.88 kg), and BMI z-score (−0.37), while controls showed minimal increases.	Vegetable intake showed significant time × group interaction (*p*-value = 0.003) Dietary quality improved, including increased fiber intake (+2.36 g/day) and reduced saturated fat consumption (−9.24 g/day).	This intervention effectively improved body composition, dietary quality, and nutrition knowledge. These findings provide evidence for implementing culturally adapted school gardening programs as childhood obesity prevention interventions.
Jaenke et al., 2012 [31]	Nutrition knowledge was not assessed in this study	Not reported	Participants in the intervention group showed greater improvements in vegetable preferences and taste ratings compared with controls (*p* < 0.05). Girls in the intervention group demonstrated greater increases in vegetable preference and taste ratings compared with boys (*p* < 0.05, interaction effect). No significant differences were observed between intervention and control groups in fruit and vegetable intake (all *p* > 0.05).	The intervention improved vegetable preferences and taste ratings, particularly among girls, but did not result in significant changes in fruit and vegetable intake. Findings should be interpreted with caution due to small sample size, short duration, reliance on self-reported intake measures, and potential clustering effects.
Jeans et al., 2023 [38]	Not reported as a primary outcome	Not reported as a primary outcome	The intervention, increase in consumption of unprocessed foods (2.3% compared with −1.8% g; *p* < 0.01) and a decrease in UPF (−2.4% compared with 1.4% g; *p* = 0.04). In addition, Hispanic children in the intervention group had an increase in unprocessed food consumption and a decrease in UPF consumption compared to non-Hispanic children (−3.4% compared with 1.5% g; *p* < 0.05).	Study results suggest that school-based gardening, cooking, and nutrition education interventions can improve dietary intake, specifically increasing unprocessed food consumption and decreasing UPF consumption.
Kearney et al., 2024 [35]	Students who participated in VCL showed notice able improvements in cooking confidence and knowledge (r = 0.55; *p* < 0.001). Confidence was correlated with consumption behavior changes (r = 0.18; *p* = 0.022). Confidence was positively associated with consumption changes in both our adjusted (OR = 1.81; *p* < 0.001) and unadjusted models (aOR = 1.88; *p* = 0.013).	NA	Most students also reported individual changes in their consumption behaviors and preferences for certain vegetables.	This research demonstrates that programs integrating practical cooking skills education along with nutrition, food, and cooking education can improve confidence and knowledge about healthy food choices amongst children driving an overall improvement in children’s eating habits.
Kim et al., 2020 [32]	Significant post-intervention improvements were observed in nutrition and gardening knowledge, self-efficacy, outcome expectancies, and vegetable preferences (all *p* < 0.001), while food neophobia decreased (*p* < 0.05). Neophobia decreased only in younger students (*p* < 0.01; older group *p* > 0.05). Positive correlations were found between key mediators, including self-efficacy with vegetable preference (r = 0.44) and outcome expectancies (r = 0.30), and gardening knowledge with vegetable preference (r = 0.23).	Not assessed	Vegetable consumption frequency significantly increased after the intervention (*p* < 0.001). Grade-level analyses confirmed significant improvements in vegetable consumption for both groups (*p* < 0.01).	The multi-component garden-based intervention significantly improved vegetable consumption and related psychosocial determinants (knowledge, self-efficacy, preferences), while reducing food neophobia. Findings should be interpreted with caution due to the absence of a control group, pre–post design, reliance on self-reported measures, and potential confounding effects.
Labbe et al., 2023 [43]	Students who participated in the program reported a greater increase in their cooking skills (*p* = 0.013) and food knowledge (*p* = 0.028) than students in the control group.	NA	No effect was found on food skills and vegetables, fruit, and breakfast consumption (*p*-values > 0.05). Boys improved their cooking skills (*p* = 0.025) and food knowledge (*p* = 0.022), but girls did not.	This study is one of the first to assess the impact of a culinary program on three major components of food literacy among boys and girls separately. Cooking skills and food knowledge of grade 4 and 5 students can be significantly improved after participating in a 6-week culinary program and boys may benefit the most. However, the program was ineffective at increasing vegetable and fruit consumption or the odds of eating breakfast regularly.
Landry et al., 2019 [39]	No significant differences were observed between intervention and control groups at baseline for psychosocial variables (*p* > 0.05).	At baseline, 51% of participants were overweight or obese, with no differences between intervention and control groups (*p* > 0.05). Changes in cooking and gardening behaviors were not associated with changes in BMI z-score or waist circumference (all *p* > 0.05).	At baseline, mean dietary intake was 1371 kcal/day, 0.96 cups/day of vegetables, and 13.7 g/day of dietary fiber, with no differences between groups (*p* > 0.05). Regression analyses showed that increases in cooking behaviors were significantly associated with increases in dietary fiber intake (*p* = 0.004) and vegetable intake (*p* = 0.03). Increases in gardening behaviors were significantly associated with increased dietary fiber intake (*p* = 0.02).	Improvements in cooking and gardening behaviors were associated with better dietary intake (fiber and vegetables), but not with anthropometric changes. Findings should be interpreted with caution due to the secondary (post hoc) analytical approach, lack of between-group effects despite randomization, reliance on self-reported dietary intake, and short intervention duration.
Landry et al., 2021 [37]	Not reported as a primary outcome	Not reported	Modest increase in protein intake as a percentage of total energy (0.4% vs. −0.3%, *p* = 0.021) and in HEI-2015 total vegetables component scores (+4% vs. −2%, *p* = 0.003). Non-Hispanic children had a significant increase in HEI-2015 total vegetable scores in the intervention group compared to the control group (+4% vs. −8%, *p* = 0.026). Both increased added sugar intake; however, to a lesser extent within the intervention group (0.3 vs. 2.6 g/day, *p* = 0.050).	School gardens can play a critical role in shifting children’s perceptions of food and enhancing their access to healthful foods
LeBlanc et al., 2022 [36]	Students in the PC course reported greater increases in food (β = 5.74, 95% CI 1.65, 9.83) and cooking skills (β = 10.33, 95%CI 5.59, 15.06) than students in the PSD course. Girls and boys in the PC course reported greater improvements in cooking skills (β = 8.68, 95% CI 2.57, 14.80; β = 11.97, 95% CI 4.39, 19.57, respectively) than those in the PSD course.	NA	Gender analyses also showed that the PC course was particularly effective at improving boys’ and girls’ cooking skills. Considering that learning how to cook during childhood or adolescence has been linked to improved dietary outcomes during adulthood, the integration of cooking courses in schools’ curricula is encouraged.	This study is one of the first to assess the effectiveness of a curriculum-based high school experiential cooking course on adolescent boys’ and girls’ cooking skills, food skills, consumption of vegetables and fruits, and other eating behaviors
Maiz et al., 2021 [44]	Improvements were observed for neophobia for the HO group and cooking self-efficacy and KidMed score for both groups.	NA	Students from the HO group selected and ate more spinach/broccoli (*p* < 0.001 and *p* = 0.02, respectively) for the first lunch; and selected more spinach/broccoli (*p* = 0.04) for the second lunch.	The interventions effectively enhanced children’s diet quality, although only the HO group decreased food neophobia levels.
Parmer SM et al., 2009 [29]	Participants in the NE + G and NE groups showed greater improvements in nutrition knowledge compared with the control group (*p* < 0.05).	Not measured/reported	Participants in the NE + G and NE groups reported higher taste ratings for vegetables compared with controls (*p* < 0.05). Additionally, the NE + G group was more likely to select and consume vegetables in the lunchroom setting at post-intervention compared with both the NE-only and control groups (*p* < 0.05).	The garden-based experiential learning program improved nutrition knowledge and food preferences and showed some positive effects on fruit and vegetable consumption. Findings should be interpreted with caution due to potential biases, including small sample size, cluster design with limited number of units, reliance on self-reported measures, and short follow-up duration.
Zahr et al., 2017 [30]	Students in the intervention group reported greater improvements in cooking skills compared with the comparison group, including cutting vegetables and fruit (97% vs. 81%), measuring ingredients (67% vs. 44%), using a knife (94% vs. 82%), and preparing a balanced meal independently (69% vs. 34%) (all *p* ≤ 0.05). Cooking confidence also increased significantly for specific recipes, including fruit salad (85% vs. 81%), minestrone soup (25% vs. 10%), and vegetable tofu stir fry (39% vs. 26%) (*p* ≤ 0.05)	Anthropometric outcomes were not assessed.	Students exposed to the intervention reported increased familiarity and preference for foods introduced in the program, with significant improvements for broccoli, Swiss chard, carrots, and quinoa (*p* ≤ 0.05). No objective dietary intake measures were assessed.	The intervention improved food preferences, cooking skills, and confidence, but findings should be interpreted with caution due to the quasi-experimental design, small sample size, short follow-up, and reliance on self-reported outcomes.

Abbreviations. BMI: body mass index; CI: confidential interval; G: gardening; HEI: healthy eating index; HO: hands-on; NA: not applicable; NE: nutritional education; OR: odds ratio; PC: Professional cooking; PSD: personal and social development; UPF: ultra processed food.

In the same study, vegetable intake showed a significant time × group interaction (*p* = 0.003) but small absolute changes [34].

Additional evidence from a randomized controlled trial by Sondra M. Parmer and colleagues demonstrated that school gardens can function as an effective experiential nutrition education strategy in young children. In this study, second-grade students participating in a garden-based intervention showed significant improvements in fruit and vegetable knowledge, preferences, and self-reported consumption compared with controls, supporting the role of hands-on gardening activities in promoting healthier eating behaviors early in life [29]. These findings further reinforce the potential of school garden programs to enhance both cognitive and behavioral nutrition-related outcomes in primary school setting.

Hands-on cooking interventions and chef-led programs mainly reported effects on proximal vegetable-related outcomes. In Spain (8–9 years), the hands-on group selected and consumed more spinach (*p* < 0.001) and broccoli (*p* = 0.02) in the first experimental lunch and selected more spinach/broccoli in the second lunch (*p* = 0.04). Post-intervention, food neophobia improved only in the hands-on group; cooking self-efficacy increased in both groups; and diet quality (KidMed) improved in both [44]. In the UK (9–11 years), a chef-led intervention reported a significant increase in vegetable consumption scores (2.24 to 2.46; Δ = +0.22) and cooking confidence (3.09 to 3.35; Δ = +0.26) relative to controls [41]. By contrast, a curriculum-based secondary school culinary course produced substantial improvements in competencies but no measurable effects on fruit and vegetable intake (all *p* > 0.05) [37]. Anthropometric outcomes were generally not reported for the cooking-focused studies.

Cognitive outcomes were among the most consistent findings. In LA Sprouts (grades 3–5), the intervention improved vegetable identification (+11% vs. +5%; *p* = 0.001) and nutrition and gardening knowledge (+14.5% vs. −5.0%; *p* = 0.003), and increased the likelihood of home gardening (+7.5% vs. −4.4%; *p* = 0.003) [42]. A garden-based integrated program (mean age 11.6 years) also reported significant increases in nutrition and gardening knowledge [32], consistent with the Jordanian intervention, which showed a substantially greater improvement in nutrition knowledge in the intervention group (+22.31 vs. +1.75; *p* ≤ 0.001) [33].

For cooking interventions, the most robust outcomes concerned cooking skills and applied competencies. Project CHEF (grades 4–5) reported increased familiarity/preference for foods introduced (including significant improvements for selected items, such as broccoli, Swiss chard, carrots, and quinoa; *p* ≤ 0.05), alongside gains in practical skills and confidence in preparing recipes [41]. A Canadian school-based program (9–10 years) improved cooking skills (*p* = 0.013) and food knowledge (*p* = 0.028) with no effect on vegetable/fruit intake or breakfast consumption (*p* > 0.05) [30]. In extracurricular programs (grades 3–11), Vetri Cooking Lab reported significant increases in cooking knowledge and confidence (*p* < 0.001), a positive knowledge–confidence association (*p* < 0.001), and an association between confidence and reported consumption behavior changes (*p* = 0.022) [35]. Overall, the evidence suggests that experiential formats reliably strengthen food literacy components related to applied knowledge and skills.

Experiential programs frequently reported improvements in psychosocial determinants and vegetable acceptability. In integrated garden-based interventions (mean age 11.6 years), increases in self-efficacy, outcome expectancies, vegetable preference and self-reported vegetable consumption, and reductions in food neophobia were reported [32]. In cooking interventions, Cooking With Kids (fourth grade) improved fruit and vegetable preferences, largely driven by vegetable preferences; effects were stronger in the cooking + tasting group and in boys (*p* = 0.045 and *p* = 0.033), and children without prior cooking experience showed larger gains in cooking self-efficacy (*p* = 0.004) and attitudes (*p* = 0.003) [29]. In the Spanish quasi-experiment, neophobia decreased only in the hands-on group, while cooking self-efficacy increased in both groups [44]. In the Australian garden-plus-curriculum study (11–12 years), improvements were mainly observed in willingness to taste and taste ratings, without significant changes in vegetable intake [31].

Within LA Sprouts, behavioral analyses further supported a potential mediating role of specific practices: increases in cooking behaviors were associated with higher fiber and vegetable intake, whereas increases in gardening behaviors predicted higher fiber intake, with no associations with anthropometric parameters [39]. This pattern is consistent with the interpretation that observed dietary changes may be driven by acquired skills and practices rather than broad attitudinal shifts alone.

### 3.4. Workshop of Camps

The school age is the period when children experience the fastest physical, cognitive, and social development. This period is characterized by forming lifelong behaviors and is the most suitable time for acquiring information and establishing habits. It is also a high-risk period for the development of adult diseases. Consequently, instilling healthy eating habits in children during school age can help them carry these habits into later life, prevent nutritional deficiencies, and ensure optimal growth and development. Consequently, this period offers a great opportunity to enhance children’s nutritional behaviors and dietary intake. Particularly schools provide a unique setting that integrates daily activity goals with learning and personal development. The educational method is also a crucial factor to consider when delivering nutrition education to school-age children.

A recent study [45] explored the impact of the workshop about the healthy plate model on the nutritional habits, dietary behaviors, and nutritional knowledge of school age children. The findings showed that school-based healthy nutrition training and workshops can positively impact dietary intake, such as eggs, legumes, vegetables, fruits, and oilseeds daily, increased after the training (*p* > 0.05), similarly the consumption of whole fruit increased from 35.3% to 47.1% after the training (*p* < 0.05). Moreover, the mean nutritional knowledge score (before: 71.37 ± 11.8; after: 80.45 ± 1.6, *p* < 0.05) and the Mediterranean Diet Quality Index (KIDMED) score (before: 4.77 ± 2.41; after: 5.50 ± 2.45, *p* < 0.05) increased significantly after the training.

Overall, the authors concluded that structured nutrition education for school-age children can positively impact their nutritional knowledge and dietary habits.

Another study in Danish school children [46] aimed to evaluate the effect of the school-based educational intervention “FOODcamp” on dietary habits among sixth–seventh graders, focusing on the food groups. No statistically significant effects of participating in FOODcamp was found on the average food intake of the four food groups eaten regularly (vegetables, fruit, vegetables/fruit/juice combined, or meat) (*p* > 0.05). Among the food groups not eaten regularly (fish, discretionary foods, and sugar-sweetened beverages), a non-significant tendency to lower odds of consuming sugar-sweetened beverages from baseline to follow-up (OR = 0.512; 95% CI: 0.261–1.003; *p* = 0.0510) was seen.

In conclusion, this study found no effect of the educational intervention FOODcamp on the dietary intake of vegetables, fruit, vegetable/fruit/juice combined, meat, fish, or sugar-sweetened beverages. The intake frequency of sugar-sweetened beverages tended to decrease among FOODcamp participants. Characteristics and results of these two studies are shown in Table 7 and Table 8, respectively.

It is crucial to integrate nutrition education into the school curriculum and incorporate the concept of adequate and balanced nutrition into children’s education to ensure optimal growth and development, as well as to instill lifelong healthy eating habits. While school-based nutrition education can enhance children’s nutritional knowledge, this alone does not always lead to healthy dietary behaviors. This fact highlights the need to explore intervention models tailored to the developmental stage of school-age children and that can promote lasting behavioral changes. Repeating intervention studies with larger samples and conducting long-term follow-ups may provide a more comprehensive understanding of this topic.

## 4. Discussion

Overall, the included studies indicate that experiential and interactive approaches to school-based nutrition education, including game-based interventions, digital programs, and hands-on activities, such as cooking and gardening, consistently improve nutrition knowledge, food literacy, and several psychosocial determinants of healthy eating [3,5,7]. Across intervention types, gains were most evident in proximal outcomes, such as understanding of food groups and dietary guidelines, cooking and food preparation skills, self-efficacy, willingness to try new foods, and reductions in food neophobia [3,11]. These findings suggest that interactive learning environments may strengthen the cognitive and motivational processes that underpin healthier food choices, supporting the concept of food literacy as a key target of school-based interventions [2,7].

Despite these consistent improvements in knowledge and psychosocial factors, effects on dietary intake and overall diet quality were more heterogeneous [5,6]. Interventions based on board games demonstrate effectiveness during the intervention period [8,10,14]; however, their long-term impact remains uncertain. Some interventions, particularly those on lunch or canteen, reported improvements in vegetable intake [12], diet quality indicators [21], or reductions in sugar consumption [14,16] and ultra-processed foods. However, several studies observed modest or null changes in overall fruit and vegetable intake. This pattern is particularly evident in gardening- and cooking-based interventions, where significant improvements in nutrition knowledge, food preferences, and psychosocial determinants are consistently reported (*p* < 0.05), but effects on actual dietary intake are often limited or non-significant [29,31,32,43]. For example, a large cluster-randomized trial of a school gardening intervention showed no significant adjusted effect on fruit and vegetable intake (−40 g/day; 95% CI −88, 1; *p* = 0.06), although higher levels of gardening implementation were associated with a significant increase in intake (+81 g/day; 95% CI 0, 163; *p* = 0.05) [40]. Similarly, cooking interventions improved food preferences, cooking skills, and confidence (*p* ≤ 0.05), and increased willingness to try new foods (*p* < 0.05), but did not consistently translate into measurable changes in dietary intake (*p* > 0.05) [29,41,43,44].

Multi-component programs combining cooking, gardening, and nutrition education appear more promising, suggesting a mediating role of experiential behaviors. However, because many included interventions were multi-component, the specific contribution of experiential education alone cannot be clearly isolated. In the LA Sprouts intervention, increases in cooking and gardening behaviors were associated with higher fiber intake (*p* = 0.004 and *p* = 0.02, respectively) and higher vegetable intake (*p* = 0.03), although no significant effects were observed on BMI z-score or waist circumference (*p* > 0.05) [39]. Similarly, integrated garden-based interventions reported significant improvements in vegetable consumption and psychosocial determinants, including knowledge, self-efficacy, and preferences (*p* < 0.001), along with reductions in food neophobia (*p* < 0.05) [32,44], although the magnitude of dietary changes remained variable.

These inconsistencies may partly reflect methodological differences across studies, including variations in dietary assessment tools (e.g., FFQs, 24 h recalls, or direct observation), reliance on self-reported data, and differences in intervention duration and follow-up periods [3,5]. In addition, dietary behavior is shaped by multiple contextual factors beyond the school environment, including family food practices, food availability at home, and broader socio-economic influences, which may limit the translation of knowledge gains into sustained behavioral change [4,5].

Evidence on anthropometric outcomes was generally limited [6]. Most interventions were relatively short and not specifically designed to influence weight-related outcomes, which may explain the absence of consistent changes in BMI or body composition. When improvements in anthropometric indicators were observed [13,16], they were typically associated with longer or more comprehensive programs that combined nutrition education with broader lifestyle components [16]. For instance, in the study by Salahshoornezhad and colleagues [13], the smartphone game was embedded within a broader intervention, including nutrition education, aerobic exercise, and cognitive-behavioral therapy, making it difficult to isolate the specific contribution of the digital component. The intervention led to a significant reduction in BMI (*p* = 0.01), along with decreases in weight (*p* < 0.001) and hip circumference (*p* < 0.001), in a specific and clinically defined population of overweight and obese girls. The eating behaviors could therefore derive from the combined effect of multiple synergistic components rather than the game-based approach alone. These findings support the view that school-based education alone is unlikely to produce measurable anthropometric changes in the short term, although it may contribute to longer-term risk reduction by improving dietary habits and food-related competencies. Notably, this population, already characterized by an altered metabolic and behavioral profile, may be more responsive to intensive, multi-component interventions, limiting the generalizability of these findings to the broader pediatric population.

Importantly, this review highlights the role of implementation quality and program integration within the school environment [5,7]. Interventions embedded within the curriculum, supported by teachers, or linked to environmental modifications, such as cafeteria changes or school gardens, tended to show stronger effects [12,20,21]. However, findings were not uniform across studies. For instance, in a UK cluster-randomized trial, teacher-led gardening was associated with higher self-reported fruit consumption (*p* < 0.009) and willingness to try new fruits (*p* = 0.045), while externally led (RHS) interventions showed greater improvements in vegetable recognition (*p* = 0.031). No significant differences were found in overall attitudes or in the association between knowledge and intake [40]. Similarly, digital interventions that combined educational content with behavioral prompts, self-monitoring tools, and personalized feedback produced more consistent behavioral improvements than those based solely on information delivery [18,23,25].

Taken together, the evidence suggests that experiential and technology-mediated approaches may enhance engagement and strengthen the skills and motivations required for healthier eating behaviors among children and adolescents [1,7]. However, translating these proximal gains into sustained dietary change likely requires multi-component strategies that extend beyond classroom education [4,5]. Greater integration between school curricula, food environments, and family involvement may therefore be essential to maximize the long-term impact of nutrition education interventions.

Future research should prioritize longer follow-up periods, objective measures of dietary intake where feasible, and evaluations of implementation processes. Understanding how experiential learning can be effectively integrated within broader school health frameworks will be critical to support the development of scalable interventions capable of improving children’s dietary behaviors and long-term health trajectories.

## 5. Conclusions

Experiential and interactive approaches to school-based nutrition education, including game-based learning, digital platforms, and hands-on activities, such as cooking and gardening, appear to be promising strategies for improving nutrition knowledge, food literacy, and several psychosocial determinants of healthy eating among children and adolescents. Across intervention types, many studies reported gains in domains, such as self-efficacy, willingness to try new foods, and practical food-related skills, suggesting that active participation and experiential learning may contribute to strengthening the cognitive and behavioral foundations of healthier dietary choices.

However, the translation of these proximal outcomes into sustained dietary change remains less consistent. While some interventions reported improvements in vegetable intake, diet quality indicators, or reductions in sugar consumption, effects on overall dietary patterns and anthropometric outcomes were generally modest or absent, particularly in short-term studies. These findings should be interpreted with caution, given the heterogeneity of study designs and the absence of a formal assessment of study quality and risk of bias, which may limit the strength of the conclusions.

Taken together, the available evidence suggests that school-based nutrition education may play an important role in strengthening food literacy and shaping early determinants of dietary behavior, particularly when delivered through engaging and experiential formats. To achieve more sustained behavioral impact, however, such interventions may benefit from being integrated within broader school health frameworks that also address the food environment, family involvement, and supportive institutional policies. Future research with more rigorous and standardized methodologies, including longer follow-up and quality appraisal, is needed to better understand the effectiveness and scalability of experiential nutrition education programs in improving children’s dietary habits and supporting the prevention of diet-related diseases across the life course.

## Figures and Tables

**Table 7:** Workshop or camp studies characteristics.

Authors, Year	Country	Study Design	Study Duration	Intervention Type	Follow-Up	Population Age	Sample Size	Control Group YES/NO (If Yes, Details)
Outzenet et al., 2023 [46]	Denmark	Cluster-based quasi-experimental controlled intervention study	1 year	Multi-component intervention approach and includes educational activities	2–5 weeks post-intervention	11–13	589	YES Control classes consisted of 6th and 7th grades from the same school that were not participating in FOODcamp
Simsek Sahin et al., 2025 [45]	Turkey	NA	12–16 weeks	Healthy Plate Model workshop (structured nutrition education)	No defined follow-up period; assessment conducted after training within study window	School-age children (mean age 10.2 ± 0.45 years)	102	No

Abbreviations. NA: not applicable.

**Table 8:** Workshop or camp studies: results and conclusions.

	Outcomes Results			
Authors, Year	Nutrition Knowledge	Anthropometric Measurements	Food Choices/ Dietary Intake	Conclusions
Outzenet et al., 2023 [46]	NA	NA	The proportion of children in the control group consuming fish increased from 47% at baseline to 52% at follow-up, and, for the intervention group, the number decreased from 47% at baseline to 42% at follow-up. Discretionary foods were eaten by 94% in the control group at baseline and 88% at follow up. In the intervention group, the numbers were 93% at baseline and 84% at follow-up. Finally, the percentage of children drinking sugar-sweetened beverages decreased in the control group from 86% to 73% and in the intervention group from 77% to 58%. A non-significant tendency to lower odds of consuming sugar-sweetened beverages from baseline to follow-up (OR = 0.512; 95% CI: 0.261–1.003; *p* = 0.0510) was seen among FOODcamp participants compared to control participants.	This study found no effect of the educational intervention FOOD camp on the dietary intake of vegetables, fruit, vegetable/fruit/juice combined, meat, fish, or sugar-sweetened beverages. The intake frequency of sugar-sweetened beverages tended to decrease among FOOD camp participants.
Şimşek Şahin et al., 2025 [45]	The mean nutritional knowledge score (before: 71.37 ± 11.8; after: 80.45 ± 1.6, *p* < 0.05) and the Mediterranean Diet Quality Index (KIDMED) score (before: 4.77 ± 2.41; after: 5.50 ± 2.45, *p* < 0.05) increased significantly after the training. A weak positive correlation was identified between pre-training nutritional knowledge scores and KIDMED index scores (r = 0.19; *p* = 0.045). Furthermore, a negative weak relationship was observed between post-training nutritional knowledge scores and meal-skipping status (r = −0.231; *p* = 0.019).	Body weight and height measurements, age-and gender-specific BMI and height percentiles	The number of students consuming eggs, legumes, vegetables, fruits, and oilseeds daily increased after the training (*p* > 0.05). Increases were observed in daily consumption of meat group and water (*p* < 0.05). While the proportion of students who consumed whole fruit with skin was 35.3% before the training, this rate increased to 47.1% after the training (*p* < 0.05).	In conclusion, it was determined that practical, sustainable nutrition training designed to meet the needs of school-age children positively affected their nutritional knowledge, adherence to the MD, and dietary habits. It is crucial to integrate nutrition education into the school curriculum and incorporate the concept of adequate and balanced nutrition into children’s education to ensure optimal growth and development, as well as to instill lifelong healthy eating habits. While school-based nutrition education can enhance children’s nutritional knowledge, this alone does not always lead to healthy dietary behaviors.

**Abbreviations. BMI: body mass index; CI: confidential interval; KIDMED: MD: mediterranean diet; NA: not applicable; OR: odds ratio.**

## Data Availability

No new data were created in this study. Data sharing is not applicable to this article.

## Supplementary Materials

The following supporting information can be downloaded at: https://www.mdpi.com/article/10.3390/nu18101610/s1.

## Abbreviations

The following abbreviations are used in this manuscript:

BMI	Body Mass Index
HEI	Healthy Eating Index
UPF	Ultra-Processed Foods
RCT	Randomized Controlled Trial
