# Supplementary material for: Learning About Healthy Nutrition by Doing: Experiential Approaches in School-Based Nutrition Education
Source: Nutrients. 2026 May 19;18(10):1610. doi: 10.3390/nu18101610 (PMC13209683; doi:10.3390/nu18101610)
Supplement: Supplementary file 1 [file nutrients-18-01610-s001.zip › nutrients-4228612-supplementary.pdf]

*Learning About Healthy Nutrition by Doing: Experiential Approaches in School-Based Nutrition Education*

**Arianna Bisogno [1,†], Ludovica Leone [1,†], Veronica D'Oria [2,*], Carlo Agostoni [1,3,*] and Martina Abodi [3,4]**

# Supplementary Material

**Search Strategy:**

("Schools"[Mesh] OR school*[Title/Abstract] OR "school-based"[Title/Abstract]) AND ("Health Education"[Mesh] OR "nutrition* education"[Title/Abstract] OR "nutrition program*"[Title/Abstract] OR "nutrition intervention*"[Title/Abstract] OR "dietary education"[Title/Abstract] OR "school nutrition program*"[Title/Abstract] OR "health promotion messaging"[Title/Abstract]) AND ("eating behaviour*"[Title/Abstract] OR "eating behavior*"[Title/Abstract] OR "eating habits"[Title/Abstract] OR "dietary intake"[Title/Abstract] OR "diet quality"[Title/Abstract] OR "dietary change*"[Title/Abstract]) AND ("Child"[Mesh] OR "Adolescent"[Mesh] OR children*[Title/Abstract] OR adolescent*[Title/Abstract] OR student*[Title/Abstract] OR toddler*[Title/Abstract])
